# The Chronic Effects of Copper and Cadmium on Life History Traits Across Cladocera Species: A Meta-analysis

**DOI:** 10.1007/s00244-018-0555-5

**Published:** 2018-09-03

**Authors:** Shlair A. Sadeq, Andrew P. Beckerman

**Affiliations:** Department of Animal and Plant Sciences, University of Sheffield, Alfred Denny Building, Western Bank, Sheffield, S10 2TN UK

## Abstract

The effect of sublethal concentrations of heavy metals on cladoceran growth and reproduction is a cornerstone of modern ecotoxicology. However, the literature contains assays across numerous concentrations, on numerous species and genotypes, and conditions are far from consistent. We undertook a systematic review of the sublethal effects of copper and cadmium concentrations on *Cladocera* spp. life history (reproduction, maturation age, and somatic growth rate). Using meta-analysis, we tested the hypothesis that the effects of increasing Cu and Cd concentrations on traits may vary by species. We also evaluated where possible whether the effect of metal concentrations on traits vary by water hardness, exposure duration, or whether the metals were delivered in aqueous solution or via food. We surveyed > 200 papers, resulting in a set of 32 experimental studies representing 446 trials where the results were presented compared with *Daphnia magna*—the most commonly assayed species. We found qualitatively similar effects of Cu and Cd on life history traits that included reduction in reproduction and somatic growth rate and delay of maturation. Cladocera species showed marked variations in their susceptibility to metals, and *D. magna* was found to be the least sensitive species to sublethal changes in reproduction. The effects were largely consistent for aqueous vs. dietary food. Water hardness, where data were available, had no detectable effect. Available data indicate that exposure duration had no effect on the toxicity of Cu but did for *D. magna* reproductive response to Cd. Our study highlights the importance of including species identity when considering toxicological testing and regulation development.

**Electronic supplementary material:**

The online version of this article (10.1007/s00244-018-0555-5) contains supplementary material, which is available to authorized users.

Anthropogenic and natural processes introduce a great deal of pollutants and stress into natural ecosystems (Mountouris et al. 2002; Demirak et al. 2006; Dedourge-Geffard et al. 2009; Martins et al. 2017). Over the past few decades, trace levels of metals have received much attention (Jing et al. 2006), because they generate negative biological effects on aquatic organisms, such as algae grazers and subsequently alter water quality. Such “sublethal” effects include changes in reproduction (Bodar et al. 1988a; Wang et al. 2009; Kim et al. 2017), somatic growth rate (Chandini 1989; Koivisto et al. 1992; De Schamphelaere and Janssen 2004a), feeding rate (Ferrando and Andreu 1993; MacWilliam and Baird 2002; De Schamphelaere et al. 2007), and respiration and metabolism (Dave 1984; Bodar et al. 1988b; Khangarot and Rathore 2003).

Copper and cadmium are widely found as pollutants in natural water systems and are well-known toxicants for aquatic invertebrates, particularly Cladocera. These chemicals continue to occupy a large portion of the research agenda on sublethal effects of metal pollution (Bellavere and Gorbi 1981; Shuhaimi-Othman et al. 2010; Fernández-Gonzáles et al. 2011). However, each element has a different mode of action (Shanker 2008). Cu is an essential element to living organisms where it is primarily involved as a co-factor in enzymatic reactions of biological processes (Bossuyt and Janssen 2004; de Oliveira-Filho et al. 2004). It is considered to be a potent toxicant only at high concentrations and works largely by disrupting digestive physiology, which is linked to energy intake and hence resources acquired for growth and reproductive activities (Barata and Baird 2000; Bui et al. 2016). Its toxicity at high concentrations derives from its redox potential where it can induce reactive oxygen species formation (ROS) that lead to oxidative stress (Stoddard and Harper 2007; Giusto and Ferrari 2014). These conditions can cause damage to biological structures, such as DNA, lipoproteins, and organelles (Letelier et al. 2005; Giusto and Ferrari 2014). Furthermore, it has been demonstrated that Cu inhibits the activity of enzymes involved in cell metabolism, such as Na^+^/K^+^-ATPase and Mg^+2^-ATPase, which are responsible for the exchange of ions across the cell membrane (Pagliarani et al. 1996; Handy et al. 2002; Katranitsas et al. 2003).

On the other hand, Cd is a nonessential element and highly toxic even at very low concentrations (Gama-Flores et al. 2006; Wang et al. 2009). The International Agency for Research on Cancer of USA (1997) classified cadmium as a Type 1 carcinogen. Exposure causes a series of changes in cellular homeostasis, such as DNA damage, acidification of the cytoplasm, and oxidative stress linked to ROS formation via elevation of lipid peroxidation in tissues (Stohs and Bagchi 1995; Soetaert et al. 2007). In addition to its role in reduction of the activity of antioxidant enzymes, such as glutathione peroxidase (GPx), catalase (CAT), and superoxide dismutase (SOD) (Waisberg et al. 2003; Wang et al. 2004; Sandrini et al. 2008). Cd also strongly impacts feeding activity which hence impairs reproduction and growth (Baird et al. 1990; Barata and Baird 2000). In freshwater systems, the concentrations associated with sublethal effects for Cu range from 0.2 to 30 μg/L (Al-Reasi et al. 2012), whereas it is lower than 0.1 μg/L for Cd (Tan and Wang 2011).

In natural environments, contaminants often are diluted and occur at low concentrations. Therefore, aquatic organisms are considered to be under risk of chronic exposure to low concentrations over long periods. To evaluate the risks associated with pollutants, chronic exposure to sublethal concentrations of a stressor is typically used to examine life history responses of different species. Furthermore, short-term toxicity tests represent an early warning indicator of toxicant impacts on aquatic organisms (Stephan et al. 1985). These ecotoxicological assays are a typical method for evaluating how an organism or a population respond to contaminants exposure (Barata and Baird 2000). The data from such assays are used to define standards for ecosystem services, such as water quality monitoring, and to define impacts on natural populations, their habitats, and ecosystems (Lopes et al. 2004; Cooper et al. 2009).

Recent work suggests that the responses to sublethal concentrations of metals may be influenced by species identity (e.g., interspecific variation) and genotype (e.g., intraspecific variation) (Barata et al. 2000; Hoang and Klaine 2007). Furthermore, metal complexity, metal toxicity, and aqueous versus dietary exposure influencing bioavailability to an organism may be important (Gusso-Choueri et al. 2012). This may be further influenced by redox potential, pH, hardness, and the amount of existing metal present in water (Long et al. 2004; Bae and Freeman 2007; Hoang and Klaine 2007).

It has been demonstrated that bioavailability and toxicity of metals to aquatic species may be affected by a number of environmental factors, such as water hardness, alkalinity, pH, and dissolved organic carbon (DOC) (Santore et al. 2001). The biotic ligand model (BLM) is a promising model to assess how the water chemistry influence the toxicity of Cu to *Daphnia magna*. This model is based on the complexation of free ions and competition with other cations (from the natural and artificial media) (Bossuyt et al. 2004; De Schamphelaere and Janssen 2004b, c; De Schamphelaere et al. 2004; Clifford and McGeer 2010). Data collected from the literature agree on the decrease of Cu toxicity as a result of hardness, pH, and dissolved organic carbon (Di Toro et al. 2001; Santore et al. 2001; De Schamphelaere et al. 2004). Furthermore, BLM has recently been developed for other metals across species of invertebrates, including *Daphnia* (Keithly et al. 2004; De Schamphelaere et al. 2006; Kozlova et al. 2009; Clifford and McGeer 2010; Esbaugh et al. 2012).

Toxicity tests on a species level (single and multiple) remain the core biological level of assessment, and it is assumed that the link between ecosystems function and taxonomic variation in the response to toxicants can offer insight into how population and ecosystems may respond (Cairns 1983; Rahbek 2005). This is crucial in the context of ecotoxicology where very few species or even taxa are used to make inference about the environmental impacts of contaminants and other stressors. Whilst species differences may seem to be “common sense,” identifying such differences should provide important insight into how the evaluation of toxicant impacts is conducted. Although environmental risk assessment routinely focuses on responses of one or several species to chemicals, little is known regarding species specificity in life history responses under short- or long-term exposure.

The cladoceran species are regarded as the ideal model for ecotoxicological tests of monitoring natural ecosystems. They have a global distribution, play a central role in the aquatic food chain, are sensitive to a vast range of pollutants, are easy to handle, have a short lifespan, and demonstrate dramatic phenotypic plasticity (Lampert 2006; Stollewerk 2010). Considerable laboratory research has documented effects of sublethal concentrations of Cu and Cd on life history traits of different species of Cladocera (Baird et al. 1990; Khangarot and Rathore 2003; Sofyan et al. 2007; Dao et al. 2017) (Fig. 1). However, to date, we are unaware of any systematic review of this literature that would allow estimation of whether the effect of increasing Cu or Cd concentration on life history varies by species, how experimental conditions affect these responses, and which species demonstrate strong or weak responses (e.g., appear resistant or sensitive).

**Fig. 1:**
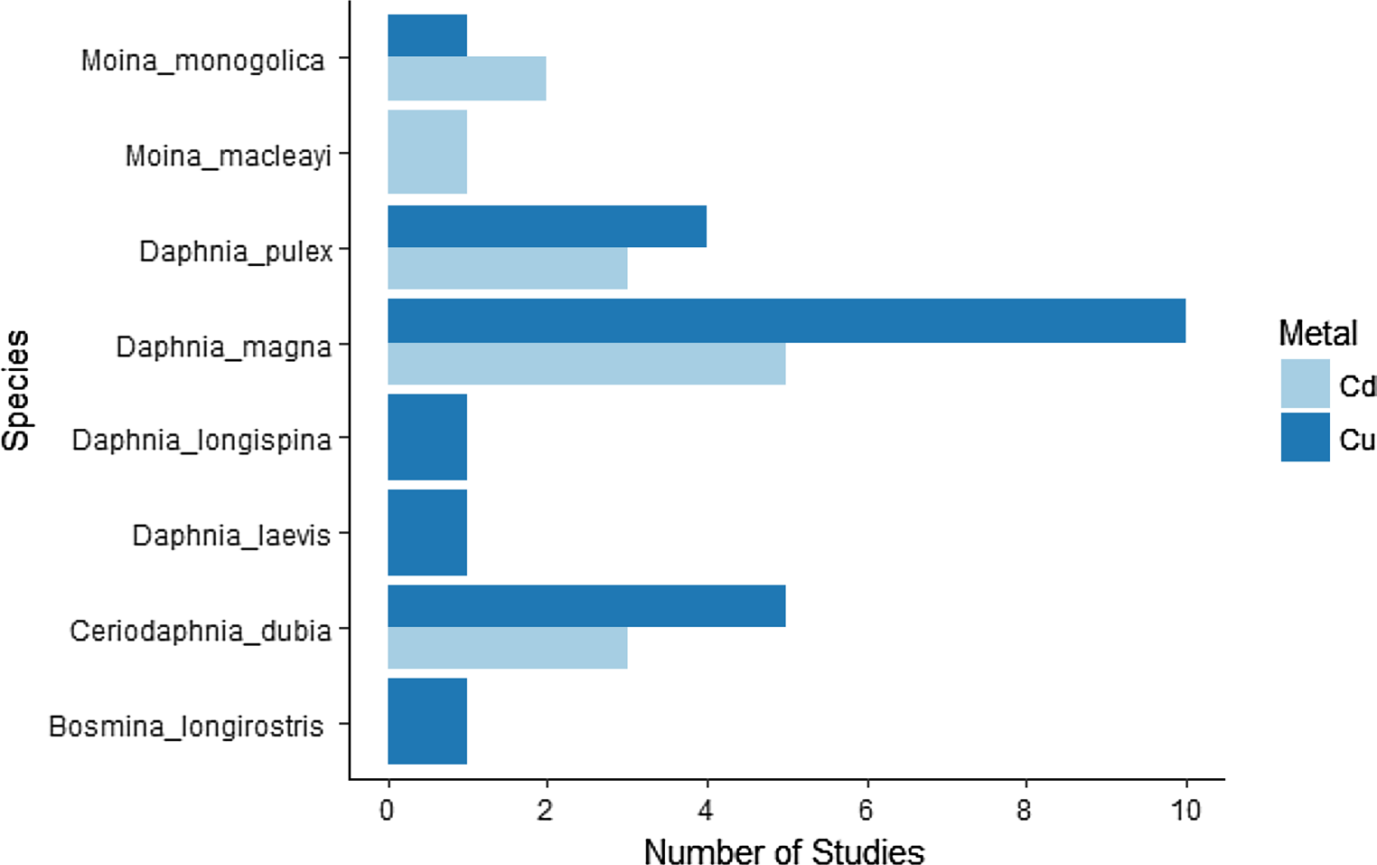
Number of studies for each species of Cladocera in response to subchronic exposure of Cu and Cd

## Expectations from Theory

Existing research centers on several aspects of *Daphnia* spp. behavior and life history: feeding rates, the number of neonates per female per day, age at maturity, and somatic growth rate. Life history theory provides a template against which the effects of Cu and Cd can be evaluated. Because they interfere with digestion, acting to limit resource intake and assimilation (De Coen and Janssen 1997; Lv et al. 2017), and ultimately interfere with metabolism (De Coen and Janssen 1998), life history theory predicts that as metal concentrations rise, foraging, and assimilation of energy may decline leading to reduced reproduction, delayed maturity, and slower somatic growth rates.

However, data reviewed on a case by case basis provide equivocal alignment with such expectations. While many published data showed that Cu and Cd have negative impacts on the production of neonates in many species (Knops et al. 2001; Luciana et al. 2014), feeding rate (MacWilliam and Baird 2002), and somatic growth rate (Agra et al. 2011), several studies also demonstrated that each metal can cause an increase in neonate production (Dave 1984; Bodar et al. 1988a; Roux et al. 1993; Agra et al. 2011).

Thus, while theory predicts marked changes in life history, there are equivocal empirical patterns among studies and species requiring meta-analytic tools to make cross study inference. The objective of our systematic review is to analyze the effects of Cu and Cd across studies, capturing variation in both species identity and specific lab conditions that might generate variation in the responses to metals.

First, we asked whether increasing Cu or Cd concentrations lead to decreased reproduction, delayed maturity and slower somatic growth rates, and whether this varies by species. Second, where possible, we tested whether aqueous versus dietary Cu and Cd induced different responses among *Daphnia* species. Finally, where possible, we considered whether lab conditions, including water hardness and exposure period, interact with Cu and Cd toxicity on life history parameters. Overall, species identity matters substantially; the most common species studied (*Daphnia magna*) appeared to be more resistant (i.e. less sensitive) to metals than other species.

## Materials and Methods

### Literature Search

We gathered evidence from the literature on the effect of sublethal Cu and Cd on the number of neonates per female per day, age at maturity, and somatic growth rate in different species of Cladocera. We searched in seven databases: Web of Science, Scopus, JSTOR, BIOSIS, ScienceDirect, Google Scholar and StarPlus (University of Sheffield library collection) for relevant publications, using the following key word combinations: (effect OR impact* OR influence*) AND (metals OR copper* OR cadmium*) AND (life history OR reproduction* OR age at maturity* OR growth rate*) AND (Daphnia OR Cladocera*). We collected studies between 1970 and 2017.

More than 200 references were obtained that measured relevant endpoints under chronic exposure of Cu and Cd. However, to be included, studies must report accessible information on the mean, standard deviation, or standard error of effects and sample sizes (Vilá et al. 2011). Publications were excluded when any of this information was missing or not estimable from the information provided. Only 32 of > 200 papers reported all data required for each trait/metal.

### Data Extraction and Effect Size Calculation

For each study, quantitative data was extracted on metal type (Cu, Cd), species identity, traits (reproduction, maturation age, and somatic growth rate), aqueous or dietary delivery of the metal, exposure duration, water hardness, and the sample size, standard deviations or standard errors, and the mean of both the control and the experimental groups. These data were obtained directly from tables or digitized from graphs or bars using Graph-Click (Arizona Software, version 3.0.3). Where necessary, we calculated the standard deviation by multiplying the standard error by the square root of the sample size.

We performed a quantitative meta-analysis focusing on whether the effect of the metals varied by species. We also explored the effects of delivery method, exposure duration and water hardness. These analyses were conducted using Osenberg et al. (1997) method allowing comparisons among studies. For each trait and trial, we calculated Cohen’s *d* according to the following equation:

1 $$ d = \frac{{X_{\text{e}} - X_{\text{c}} }}{s}J $$

where *X*_c_ is the mean of the control group, *X*_e_ is the mean of the experimental group and *s* is the pooled standard deviation of the control and experimental groups, and *J* is the corrector for bias.

2 $$ s = \sqrt {\frac{{\left( {N_{\text{e}} - 1} \right)\left( {S_{\text{e}} } \right)^{2} + \left( {N_{\text{c}} - 1} \right)\left( {S_{\text{c}} } \right)^{2} }}{{\left( {N_{\text{e}} + N_{\text{c}} - 2} \right)}}} $$

3 $$ J = 1 - \frac{3}{{4\left( {N_{\text{e}} - N_{\text{c}} - 2} \right) - 1}} $$

*S*_e_ is the standard deviation of the experimental group, *S*_c_ is the standard deviation of the control group, *N*_e_ is the number of cases of the experimental group, and *N*_c_ is the number of cases of the control group.

The primary goal of our study was to identify the species specificity of the effects of nominal concentrations of Cu and Cd on reproduction, maturation age, and somatic growth rate. Where possible, we evaluated whether an aqueous versus dietary source of food influenced this pattern and whether water hardness and exposure period influenced this pattern. To assess the overall significance of an interaction between concentration and species OR concentration and condition, we fit random effects models using maximum likelihood via the Metafor package in R (Viechtbauer 2010; R Core team 2013) version 1.1.453. We performed a likelihood ratio test (LRT) between a model with and without the interaction between metals and species to test formally whether the effect of Cu and Cd on traits varied by species/experimental conditions. Regression coefficients were then evaluated on the model using restricted maximum likelihood. Furthermore, *D. magna* was the most common species reported in the literature, and we present specific inference about the interactions (or lack of) via comparison to the *D. magna* response (reference species).

## Results

We first report whether the effect of aqueous Cu and Cd concentration on traits depend on species. This includes comparing aqueous versus dietary delivery. We then report on the experimental factors of water hardness and exposure duration.

### Copper-Reproduction: Aqueous and Dietary Delivery

Of the 12 independent studies reporting on aqueous Cu concentration and reproduction, 95% reported a nominal concentration between 0 and 120 µg/L. A total of 161 trials provide data on effect of Cu concentration on reproduction among four species of Cladocera: *Daphnia magna*, *D. longispina, Moina monogolica*, and *Ceriodaphnia dubia*.

**Fig. 2:**
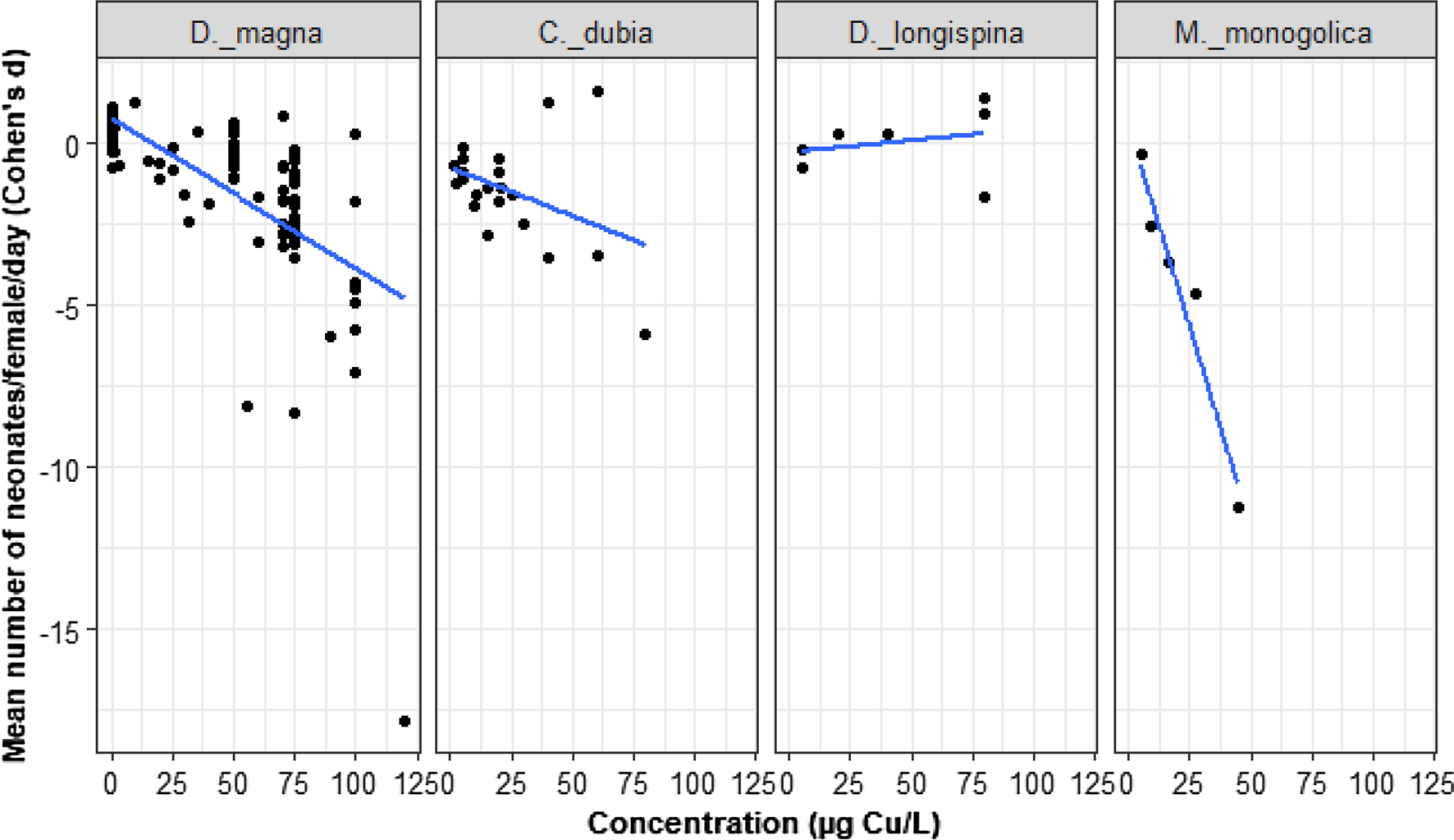
Mean number of neonates produced per female per day for four species of Cladocera exposed to ≤ 120 µg/L (nominal aqueous concentrations of Cu). The black dots represent *d* values, *n* = 161. All species are compared to the reference species (first panel). Only *D. magna* has a range of Cu concentrations up to ≤ 120 µg/L

Overall, increasing Cu concentration led to a decrease in the mean number of neonates produced per female per day and the effects of copper on reproduction varied by species (Fig. 2; LRT = 19.76, *p* = 0.0002). As Cu concentration increased, *D. magna* reproduction declined (*D. magna* slope = − 0.04, *z* = − 8.8, *p* = 0.0001, intercept = 0.64, *z* = 2.6, *p* = 0.009). The effect of Cu on *C*. *dubia* was indistinguishable from *D. magna* (gradient change from *D. magna* = 0.015, *z* = 0.95, *p* = 0.34). By comparison, Cu had a weaker effect on *D. longispina* (gradient change from *D. magna* = 0.05, *z* = 2.8, *p* = 0.005), reflecting a slightly positive effect of Cu for this species. Cu had an even stronger negative effect on *M. monogolica* (gradient change from *D. magna* = − 0.19, *z* = −3.3, *p* = 0.001).

**Fig. 3:**
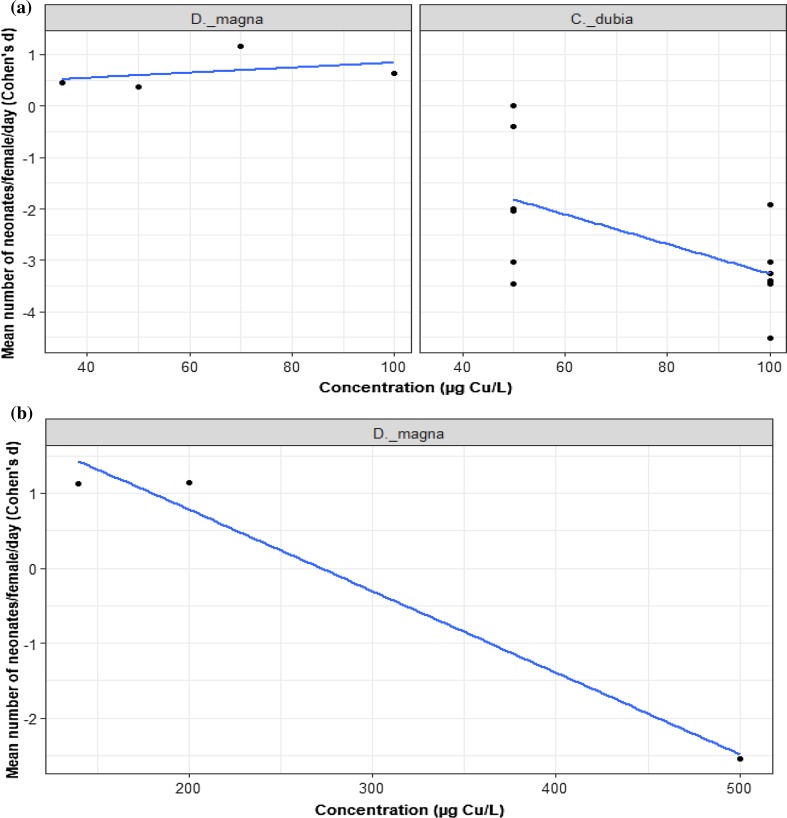
Mean number of neonates produced per female per day for **a** two species of Cladocera exposed to ≤ 120 µg/L dietary Cu (nominal), the black dots represent *d* values, *n = *16, **b***D. magna* exposed to > 120 µg/L dietary Cu (nominal), *n = *3. All species are compared to the reference species (*D. magna*)

In the subset of data that documents the effect of dietary Cu (≤ 120 µg/L,), 19 trials present data across three studies of two species of Cladocera: *D. magna* and *C. dubia*. We found that the effect of Cu concentration on reproduction does not vary by species (Fig. 3a; LRT = 2.66, *p* = 0.102). Cu increased *D. magna* reproduction (*D. magna* intercept = 1.96, *z* = 2.21, *p* = 0.027), but the concentration had no effect (*D. magna* slope = − 0.02, *z* = − 1.82, *p* = 0.067). The effect of Cu on *C. dubia* was to reduce reproduction on average (difference in intercept to *D. magna* = 2.86, *z* = 4.54, *p* = 0.0001). For dietary Cu, concentrations ≥120 µg/L had a strong negative effect, with data only available for *D. magna* reproduction, strongly driven by a single trial (from one study) at 500 µg/L Cu (Fig. 3b).

### Copper-Age at Maturity

**Fig. 4:**
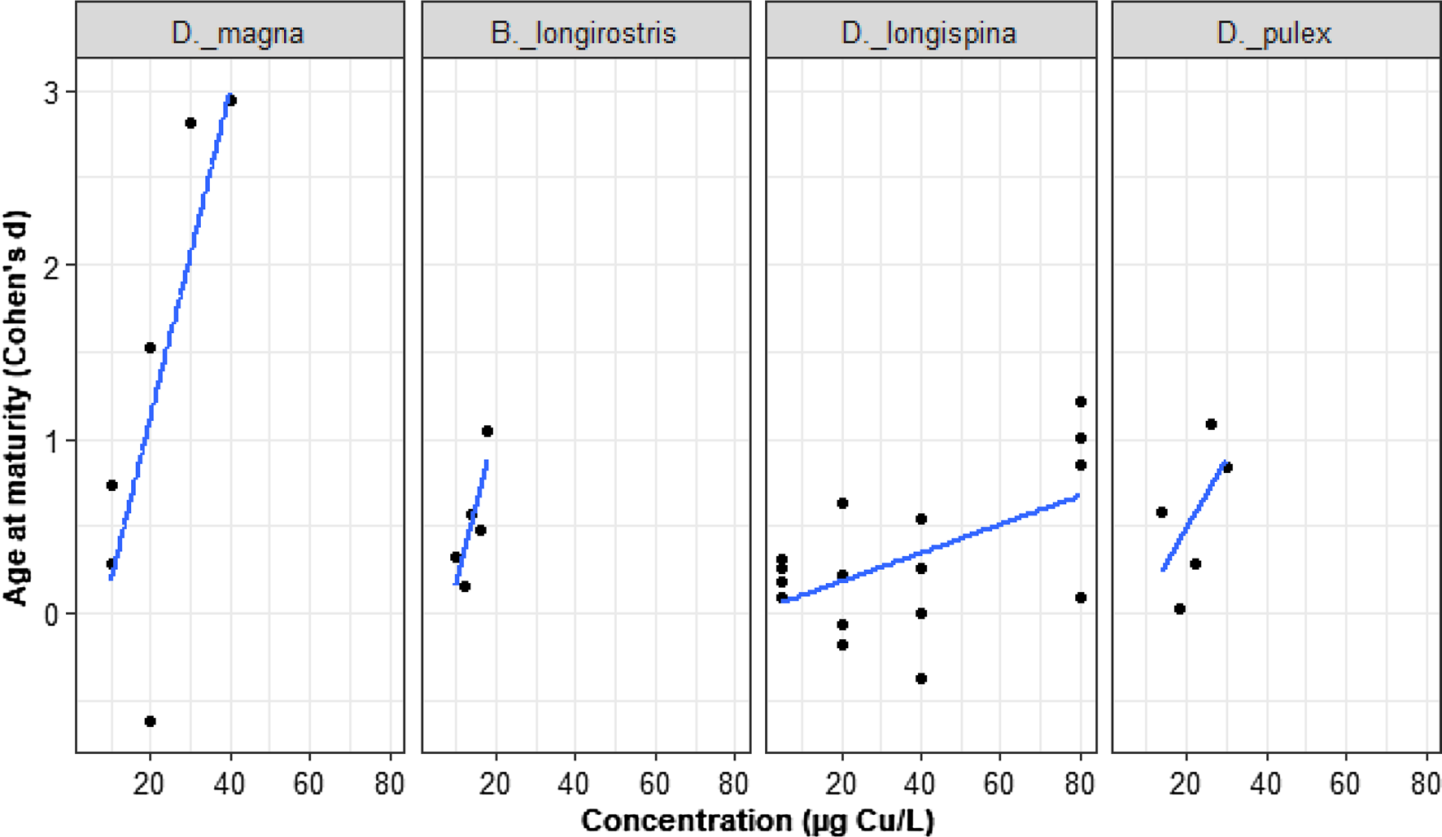
The maturation age response of four species of Cladocera exposed to nominal sublethal concentrations of Cu. The black dots represent *d* values, *n = *41. All species are compared to the reference species (*D. magna*)

Our meta-analysis for age at maturity included 41 trials of four independent studies. All data are referenced to reported nominal Cu concentrations and involve four species: *D. magna, D. pulex, Bosmina longirostris,* and *D. longispina*. Increasing copper concentration delayed maturation and this effect varied by species (Fig. 4; LRT = 9.56, *p* = 0.022). For *D. magna,* increasing Cu concentration delayed the maturation age (*D. magna* slope = 0.08, *z* = 3.10, *p* = 0.001, intercept = − 0.66, *z* = − 1.22, *p* = 0.022). The effects of Cu concentration on maturation age for both *D. pulex* (gradient change from *D. magna* = − 0.04, *z* = − 0.77, *p* = 0.436) and *B. longirostris* (gradient change from *D. magna* = 0.005, *z* = 0.06, *p* = 0.951) were not different from *D. magna*. By contrast, increasing Cu concentration delayed less severely the maturation age of *D. longispina* (gradient change from *D. magna* = − 0.07, *z* = − 2.77, *p* = 0.005).

### Copper-Somatic Growth Rate

**Fig. 5:**
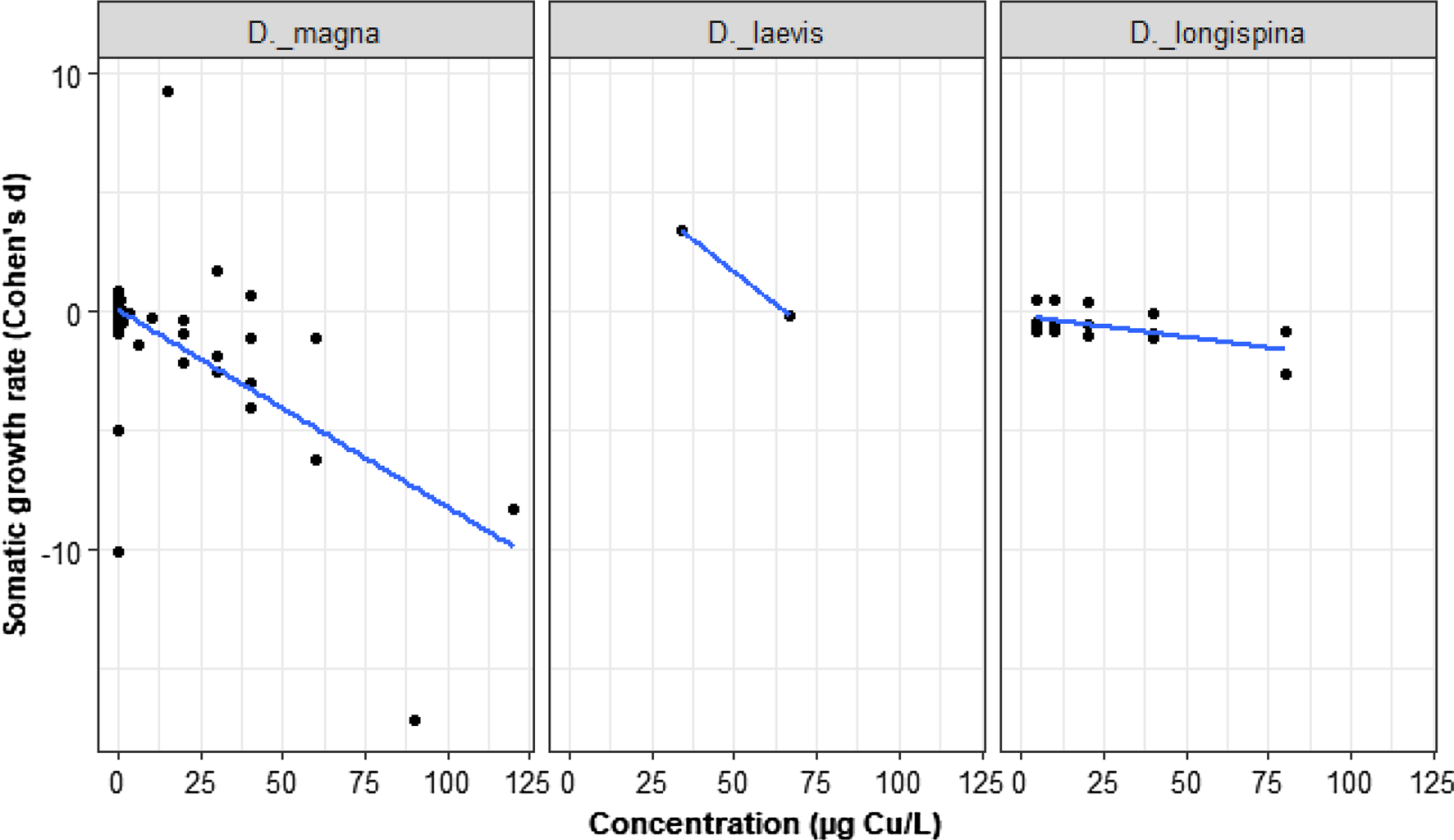
The somatic growth rate response of *Daphnia* spp. to sublethal concentrations of Cu (nominal). The black dots represent *d* values, *n = *69. All species are compared to the reference species (*D. magna*)

Our meta-analysis of somatic growth rate involved 7 studies providing 69 trials among 3 species: *D. magna, D. longispina,* and *D. laevis*. The effect of Cu concentration on somatic growth rate depends on species (Fig. 5; LRT = 8.26, *p* = 0.016). With increasing Cu concentrations, the somatic growth rate decreased for *D. magna* (*D. magna* slope = − 0.07, *z* = − 6.7, *p* = 0.0001, intercept = 0.17, *z* = 0.61, *p* = 0.54). The effect of Cu concentration on the somatic growth rate of *D. laevis* was not different from *D. magna* (gradient change from *D. magna* = − 0.03, *z* = − 0.47, *p* = 0.64). The effect of Cu concentration also reduced the *D. longispina* somatic growth rate but significantly less than for *D. magna* (gradient change from *D. magna* = 0.053, *z* = 2.72, *p* = 0.006).

### Cadmium-Reproduction: Aqueous and Dietary Delivery

Seventy-two trials from ten studies were available to explore the effects of Cd on reproduction in three species: *D. magna*, *M. monogolica,* and *C. dubia.* Ninety-five percent of the studies reported nominal concentration between 0 and 40 µg/L Cd.

**Fig. 6:**
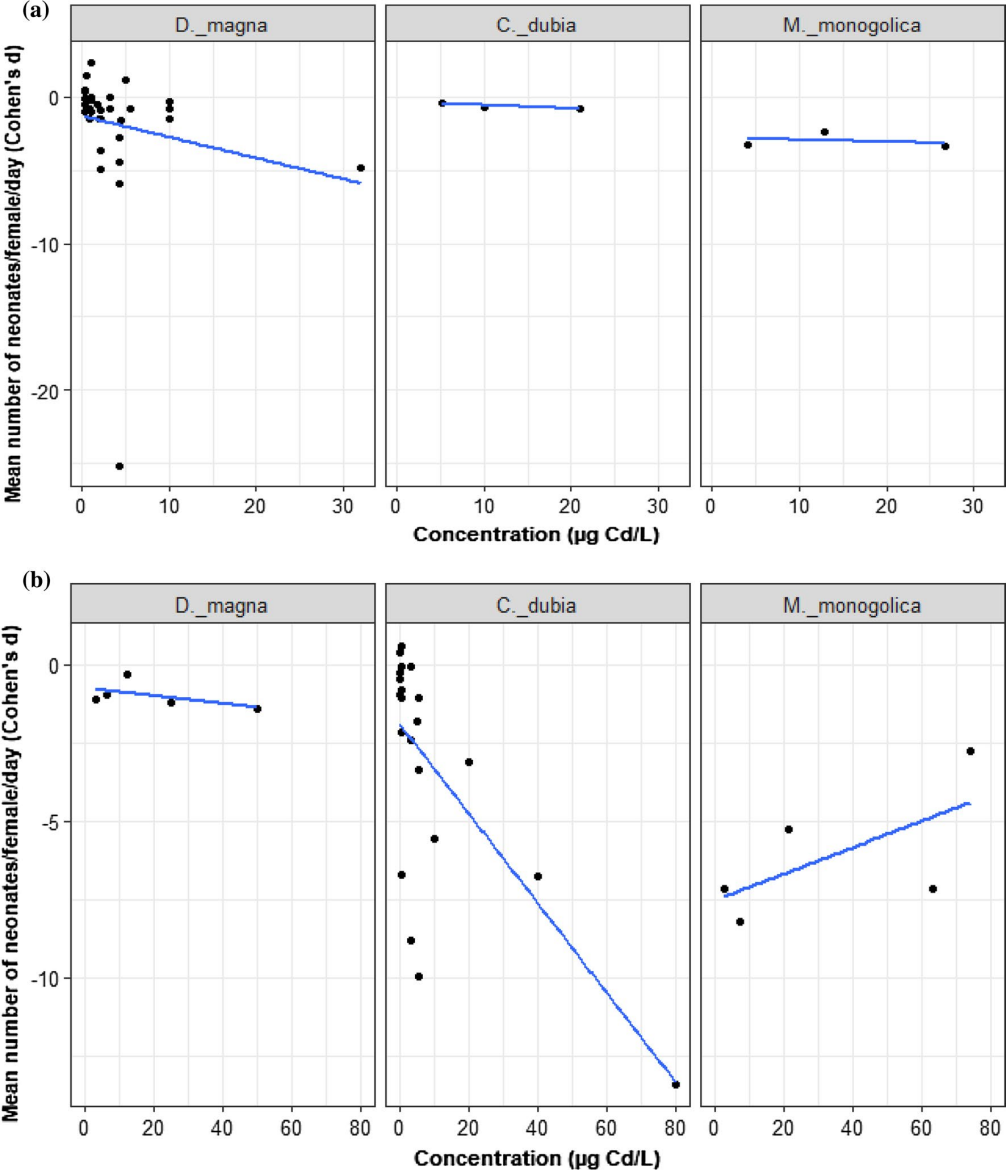
Mean number of neonates produced per female per day of three species of Cladocera exposed to **a** ≤ 40 µg/L nominal aqueous concentrations of Cd, *n = *40, **b** > 40 µg/L nominal dietary concentrations of Cd, *n = *32. The black dots represent *d* values. All species are compared to the reference species (*D. magna*)

For aqueous Cd delivery, we analyzed 40 trials from 6 studies. Increasing Cd concentration reduced reproduction, but this did not vary by species (Fig. 6a; LRT = 2.39, *p* = 0.30). As Cd concentration increased, *D. magna* reproduction decreased (*D. magna* slope = − 0.097, *z* = − 2.84, *p* = 0.004, intercept = − 0.50, *z* = − 2.02, *p* = 0.043). The average effects of Cd on reproduction on *C. dubia* (difference in intercept to *D. magna* = 1.04, *z* = 1.43, *p* = 0.150) and on *M. monogolica* (difference in intercept to *D. magna* = − 1.07, *z* = − 1.16, *p* = 0.242) were also not distinguishable from *D. magna*.

For the dietary Cd data, we analysed 32 trials from four studies of three species: *D. magna, C. dubia,* and *M. monogolica*. The effect of dietary Cd concentration on reproduction depended on species (Fig. 6b; LRT = 13.52, *p* = 0.0012). Increase of dietary Cd concentrations led to no detectable reduction in *D. magna* reproduction (*D. magna* slope = − 0.012, *z* = − 0.18, *p* = 0.855, intercept = − 0.76, *z* = − 0.44, *p* = 0.66). Cd concentration also did not reduce *C. dubia* reproduction compared with *D. magna* (gradient change from *D. magna* = − 0.13, *z* = − 1.78, *p* = 0.074; note influential data point at 80 µg/L Cd). The effect of Cd concentration on *M. monogolica* also was not different from *D. magna* (gradient change from *D. magna* = 0.05, *z* = 0.72, *p* = 0.47). However, the effect of Cd concentration on *C. dubia* was very different to *M. monogolica* (gradient change *C. dubia* to *M. monogolica* = 0.18, *z* = 3.57, *p* = 0.003). This difference drives the interaction between Cd concentration and species.

### Cadmium-Age at Maturity

**Fig. 7:**
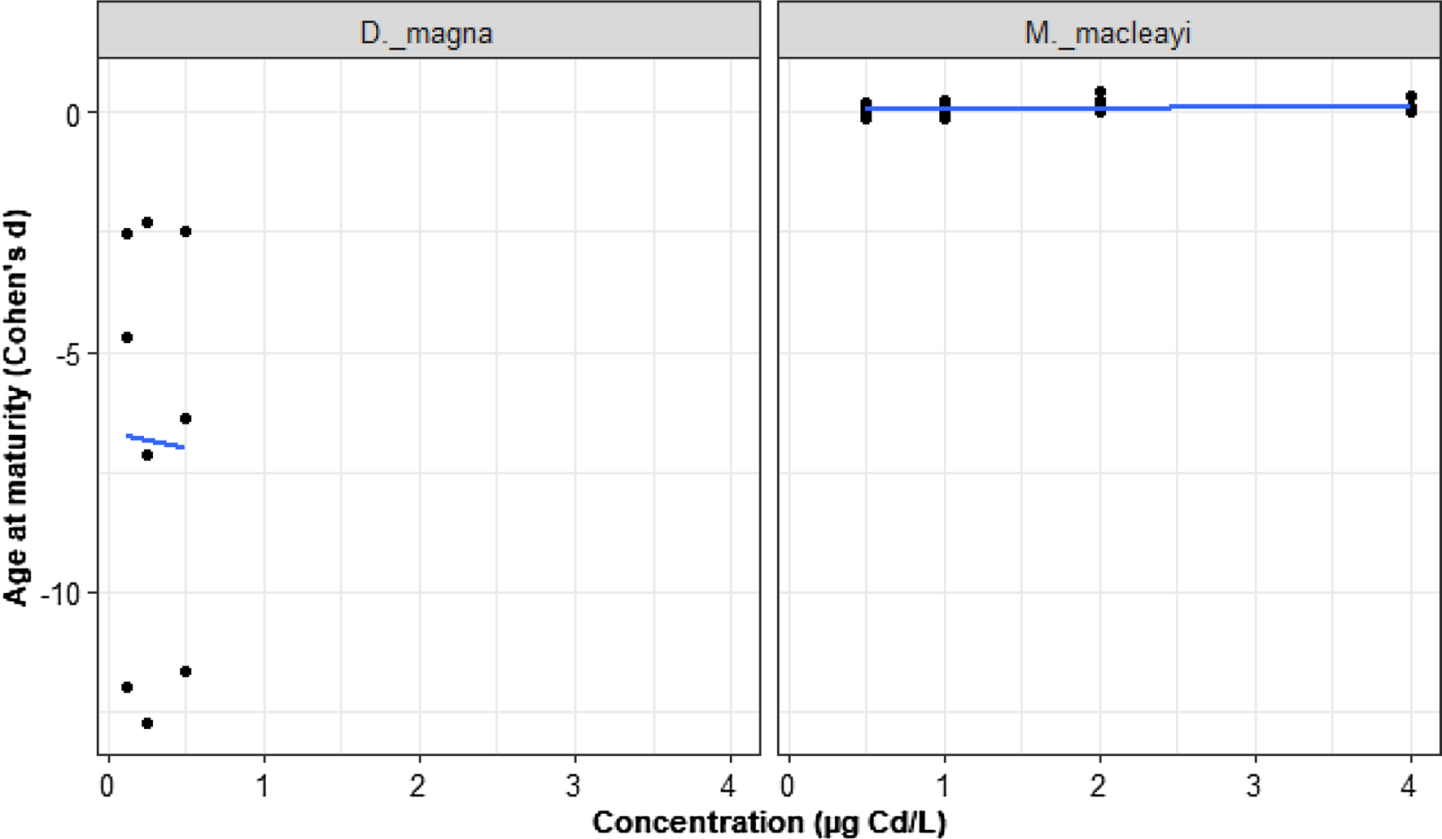
Sublethal effect of nominal Cd on maturation age of species of Cladocera. The black dots represent *d* values, *n = *41. Species are compared to the reference species (*D. magna*)

Data on Cd and maturation age came from two studies representing 41 cases in two species: *D. magna* and *M. macleayi.* The effect of Cd concentration on age at maturity did not depend on species (Fig. 7; LRT = 0.17, *p* = 0.68). For *D. magna,* Cd delayed on average the maturity age (*D. magna* intercept = − 5.69, *z* = − 11.82, *p* = 0.0001), but the concentrations had no effect (*D. magna* slope = 0.013, *z* = 0.08, *p* = 0.93). However, for *M. macleayi*, Cd did not delay the maturation age (difference in intercept to *D. magna* = 5.75, *z* = 9.91, *p* = 0.0001; *M. macleayi* has an intercept of 0).

### Cadmium-Growth Rate

**Fig. 8:**
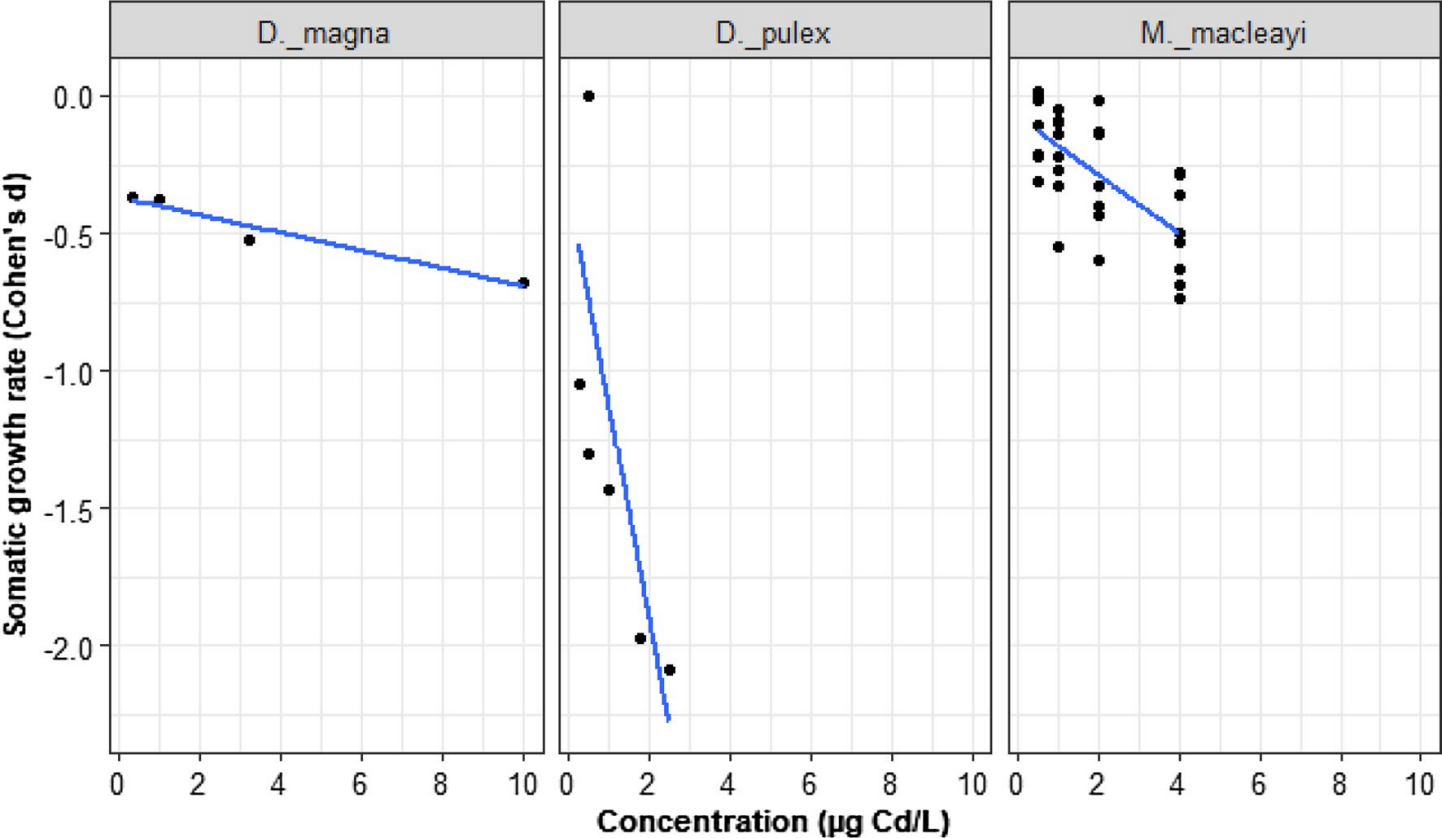
The mean somatic growth rate for *D. magna, D. pulex,* and *M. macleayi* in response to nominal Cd. The black dots represent *d* values, *n = *43. All species are compared to the reference species (*D. magna*)

The data on the effects of Cd on somatic growth rate came from four studies representing 43 trials of three species: *D. magna*, *D. pulex,* and *M. macleayi.* The effect of Cd concentration on somatic growth rate varied by species (Fig. 8; LRT = 10.77, *p* = 0.0046). There was no effect of Cd concentrations on *D. magna* somatic growth (*D. magna* slope = − 0.03, *z* = − 0.38, *p* = 0.71, intercept = − 0.37, *z* = − 0.83, *p* = 0.41). The effect of Cd on growth in *M. maclaeyi* was indistinguishable from *D. magna* (gradient change from *D. magna* = − 0.07, *z* = − 0.82, *p* = 0.408). The negative effect of Cd concentration on *D. pulex* was substantially stronger than on *D. magna* (gradient change from *D. magna* = − 0.88, *z* = − 3.26, *p* = 0.001).

### Copper and Cadmium-Water Hardness

**Fig. 9:**
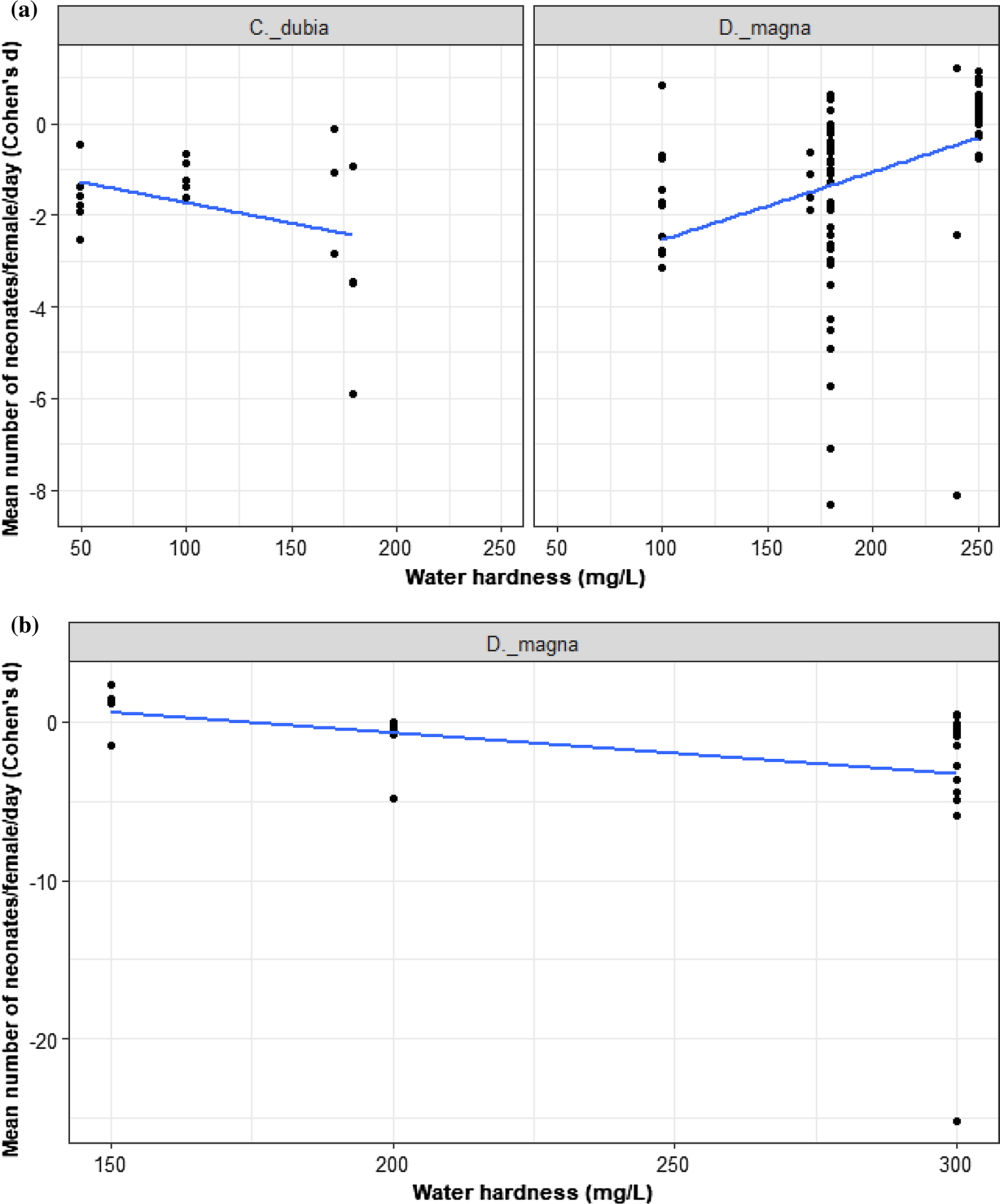
Water hardness effect on the toxicity of **a** Cu, *n = *130; **b** Cd, *n = *30 for Cladocera species reproduction. The black dots represent *d* values where species are compared with the reference species (*D. magna*)

According to the literature, water hardness levels (WH) (i.e., soft, moderate and hard) range between 40 and 300 mg/L as calcium carbonate (CaCO3). We asked for these species and trials whether the interaction between Cu or Cd concentration and species varied by WH. We constructed four models to evaluate this hypothesis. Model 1 was our full model with main effects of WH, species and metal, 2-way interactions, and the 3-way interaction. Model 2 removed the 3-way interaction. Model 3 removed both the 3-way interaction and the 2-way interactions with WH. Model 4 was the core model from all analyses above that include only the metal, species, and metal*species interaction (i.e., no effect of WH). Comparing M1 to M2 via a LRT tested the 3-way interaction. If this was not significant, we compared M2 to M3 via a LRT to test for interactions between WH and Cu or WH and species. In the absence of these interactions, comparing M3 to M4 tested for a simple additive effect of WH. We found no evidence to support the effect of WH on the interaction between Cu and species (7 studies representing 130 trials and 2 species; *D. magna* and *C. dubia*; M3 vs. M4: LRT = 2.44 and *p* = 0.12; Fig. 9a). We found no evidence to support the effect of WH on the interaction between Cd and species (3 studies and 30 trials, *D. magna* only; M3 vs. M4: LRT = 2.73, *p* = 0.097; Fig. 9b).

### Copper and Cadmium-Exposure Duration

**Fig. 10:**
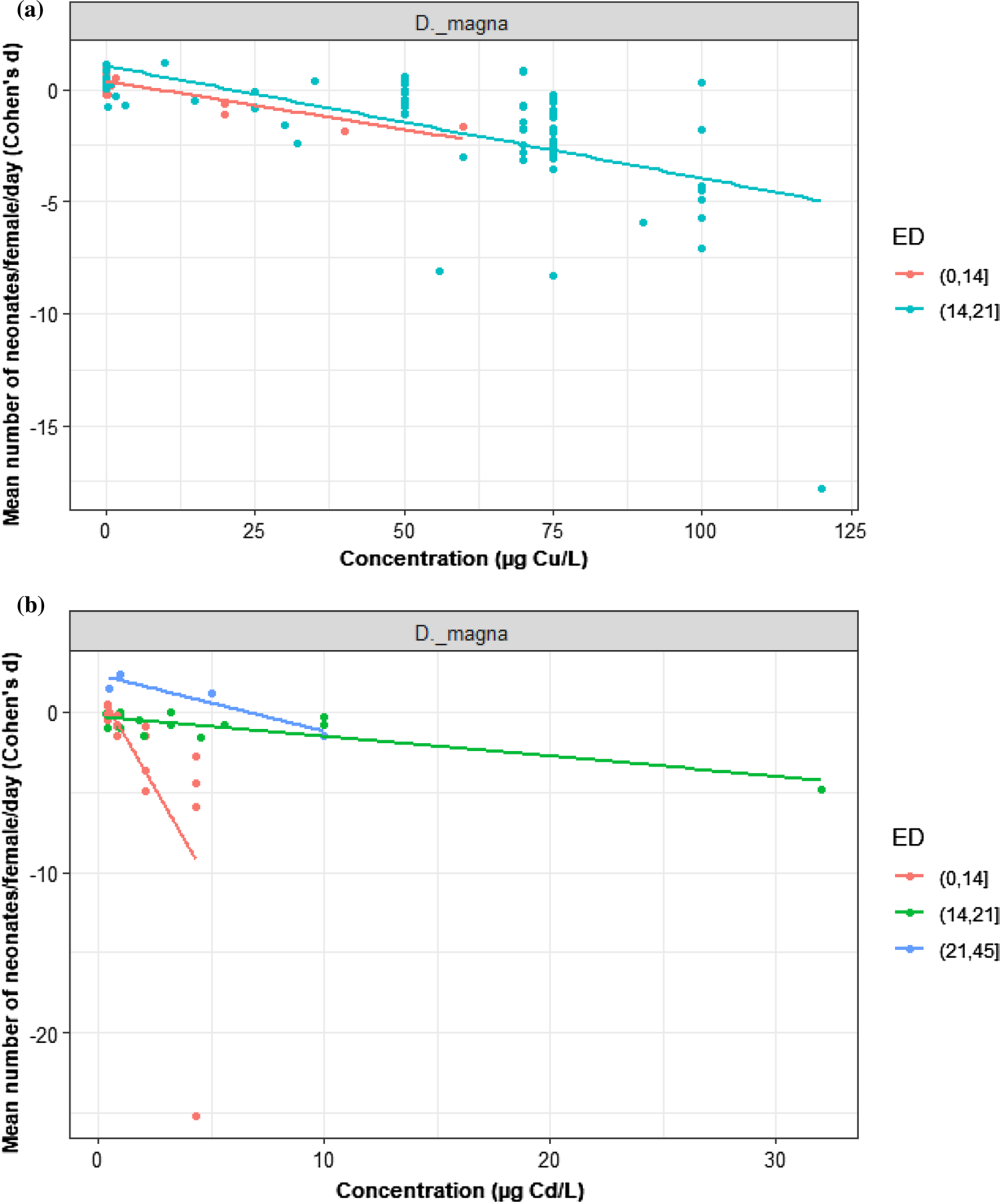
Effect of exposure duration (ED) on the toxicity of **a** Cu, *n = *113; **b** Cd, *n = *34 for *D. magna* reproduction. The black dots represent *d* values where exposure duration compared with the reference duration 14-21

We analysed 7 studies and a total of 113 trials that quantified the impact of exposure duration and Cu on reproduction. Data were only available on *D. magna* exposed to sublethal concentrations of Cu over two exposure periods: 0–14 and 14–21 (our reference duration) day trials. We found that the effect of Cu concentration on *D. magna* reproduction did not differ by test duration (Fig. 10a; LRT = 0.001, *p* = 0.97). The 21-day exposure had no effect on the toxicity of Cu (slope = − 0.04, *z* = − 7.99, *p* = 0.11, intercept = 0.51, *z* = 1.1, *p* = 0.27). The effect of Cu on reproduction over 14 days was not different from the reference duration (difference in intercept to the reference = 0.36, *z* = 0.99, *p* = 0.32).

In contrast, 4 studies represented 34 trials that quantified the impact of exposure duration and Cd concentration on reproduction. Three test periods were examined for *D. magna:* 0–14, 14–21 (our reference duration), and 21–45 days exposure. The effect of Cd concentration on *D. magna* reproduction varied by test duration (Fig. 10b; LRT = 29.17, *p* = 0.0001). Increasing Cd concentration decreased reproduction over 21 days of exposure (slope = − 1.08, *z* = − 5.38, *p* = 0.0001). The 45-day exposure was indistinguishable from the reference (gradient change to the reference = 0.73, *z* = 3.41, *p* = 0.0007). However, 14-day exposure was not affected the toxicity of Cd (gradient change to the reference = 0.28, *z* = 0.77, *p* = 0.44).

## Discussion

Cladocerans, and particularly *Daphnia* species, are classic test organisms for evaluating the sublethal effects of metals, which globally represent a serious problem to the aquatic food chain and water quality (Sarma and Nandini 2006; Altshuler et al. 2011; Martins et al. 2017). The sublethal levels of metals have received a great deal of attention, because they cause a range of ecotoxicological effects on those species. However, the importance of species specificity in understanding responses to contaminants remains challenging. Therefore, the main objective of this analysis was to assess quantitatively whether the effects of Cu and Cd on life history traits varies among species of Cladocera. Because *D. magna* is the most common test species, we were able to evaluate by direct comparison whether other species are more or less sensitive to Cu and Cd (e.g., whether *D. magna* can be considered representative). Furthermore, the selected parameters for this analysis—reproduction and somatic growth rate—are considered to be sensitive to sublethal exposure of contaminant.

Many laboratory reports have showed that the effect of contaminants or other stressors vary across species of aquatic organisms (Brix et al. 2001; Griffitt et al. 2008). However, this analysis is the first to have assessed the ecological effects of Cu and Cd across different species of Cladocera—the dominant test species in environmental monitoring and ecotoxicology. Recent meta-analysis studies have assessed the effect of contaminants on different biological levels, including species. For example, O’Brien and Keough (2014) suggest that we may expect substantial variation in life history response responses to a range of contaminants, driven by identity of contaminant, the identity of organism (taxa), and the scale of biological organization at which assessment is made (individuals, population, and community). A meta-analysis using 216 studies on the effect of toxic pollutants on marine communities also detected a strong reduction in species richness (Johnston and Roberts 2009). Furthermore, Jan Hendriks et al. (2005) and Blanar et al. (2009) detected via meta-analysis that the response to aquatic pollutants vary by population and community levels (crustaceans and parasites).

### The Influence of Metals on Life History Traits

Our systematic review supports theory on the sublethal effect of Cu and Cd across traits/species. The analysis demonstrated that overall Cu and Cd reduce reproduction, increase the age at maturity, and reduce growth, as expected by theory linked to their mode of action. However, there was substantial variation among species and *D. magna* was not uniformly the most responsive species.

We found that the effect of Cu on reproduction (only aqueous delivery), maturation age, and somatic growth rate depended on species identity. Likewise, the effects of Cd on reproduction (only dietary delivery) and somatic growth rate also depend on species identity. In many cases, the dominant test species *D. magna* often was quite resistant to nominal metal concentrations up to 120 µg/L Cu and 40 µg/L Cd. Furthermore, *D. longispina* reproductive response to aqueous Cu and *D. magna* response to dietary Cu were positive. Our data indicate that reproduction is likely the most sensitive endpoint showing strong responses to both metals among species. Life history endpoints may respond differently to the same stressor or a combination of stressors. For example, Bednarska et al. (2009) found that the reproduction of adult ground beetles was most sensitive to nickel concentrations at low and high temperatures (10 and 25 °C), but the survival was less affected by the combined effect of Ni and chlorpyriphos at both temperatures. Furthermore, Laskowski et al. (2010) conducted a meta-analysis on the interactions between toxic chemicals and natural environmental factors across a range of vertebrates and invertebrates species (including metals and *D. magna*), finding that the effects of toxicants on organisms may differ depending on external factors. Whilst, Vijver et al. (2011) suggested via meta-analysis that the effect types (additive, antagonistic, and synergistic) were significantly different across toxicological endpoints and combinations of Cu, Cd, and Zn.

Our quantitative review highlights that species vary in their sensitivity to metals. This can arise for several reasons. Mode of actions vary, the effects of pollutants can be regulated through different physiological pathways that may be species-specific, and we also expect variation due to choice of genotype used in the studies (Baird et al. 1990; Brix et al. 2001; Barata et al. 2004; Bossuyt and Janssen 2005). While it was impossible in our analysis of published data to deduce whether the same or different genotypes were being used, within species genetic variation is also likely important to consider at population and community levels. Overall, the limited number of studies reporting appropriate information for meta-analyses (sample size, means and standard deviation/error) (Furukawa et al. 2006), combined with missing detail on genotype identity, means that there is ample opportunity to pursue more rigorous assessment of these sources of variation and their impact on specificity of responses.

### The influence of the experimental factors on individuals’ responses to metals

Given natural variation in pH, water hardness, and other environmental factors in ponds and lakes, and the theoretical expectation that these factors may influence bioavailability, we also explored whether variation in experimental conditions influenced experimental effects of Cu and Cd concentrations. According to data available in this analysis, for both metals, variation among trials did not appear to arise from variation in water hardness. This correspond with the study of De Schamphelaere and Janssen (2004b) who found that water hardness had no effect on the chronic toxicity of Cu in *D. magna.* Keithly et al. (2004) showed that chronic toxicity of Ni was less dependent on hardness in *C. dubia* than acute toxicity. Our results showed that test duration had no pronounced effects on the chronic toxicity of Cu, suggesting perhaps rapid acclimation. However, under Cd exposure, *D. magna* reproduction was affected by test duration.

Our data on water hardness contrasts with what may be suggested by simply looking at results (vote counting) from the published literature. Many consider water hardness to be a crucial factor affecting metals toxicity; many reports showed that the high levels of hardness may decrease the toxicity of Cu and Cd (Winner 1986; Heijerick et al. 2003; Wang et al. 2016). However, other works indicated that heavy metals are more toxic in soft water (Ebrahimpour et al. 2010; Taylor et al. 2000). Furthermore, the effect of water chemistry on metals toxicity may be varied across the acute and chronic exposures (De Schamphelaere and Jansen 2004c). For example, Belanger and Cherry (1990) found that pH had negligible influences on the toxicity of Cu to reproduction of *C. dubia*. However, increasing pH caused a decrease in the acute toxicity (48-h mortality). Moreover, the effect of water chemistry variables on metals toxicity may show different responses under the same experimental conditions. Kozlova et al. (2009) found that sodium, potassium, and chloride ions did not affect the toxicity Ni on *D. pulex,* whereas pH effect on Ni toxicity varied in presence of HCO_3._ Similarly, the study of De Schamphelaere and Janssen (2004c) showed that dissolved organic carbon (DOC) and pH had a significant impact on chronic toxicity of Cu to *D. magna*, but water hardness did not.

Meta-analytic methods provide substantially more reliable insights into the effects across studies. While our results show limited effects, it is important to recognize that our data are a subset of all studies that report sufficient information to include in meta-analyses. A more thorough and standardized reporting of results linked to water hardness and test duration is thus warranted. Our analysis data is not always in line with literature, but the BLM and better availability of data for meta-analyses in the future will help reconcile these issues.

## Conclusions and Recommendations

This analysis is the first meta-analytic consideration of the ecological effects of Cu and Cd concentration across different species of Cladocera. The data highlight several species’ specific responses to the sublethal concentrations of both metals and several traits that, on average, appear tolerant to metals in some species. The substantial omission of numerous studies due to incomplete reporting of means, standard deviations/errors, and sample size is sobering given the importance of drawing generalized conclusions from test species. Detailed meta-analyses (and associated effective reporting of data) on water quality parameters, such as hardness, pH, and dissolved organic components (DOC), are needed to elucidate the role of water chemistry on the toxicity of metals across different biological organizations.

## Electronic supplementary material

Below is the link to the electronic supplementary material.

Supplementary material 1 (DOCX 143 kb)

Supplementary material 2 (XLSX 47 kb)

## Data Availability

Supplementary data associated with this article can be found at https://figshare.com/s/e8cd00ea26b6446fc398.
