# Supplementary material for: The Chronic Effects of Copper and Cadmium on Life History Traits Across Cladocera Species: A Meta-analysis
Source: Arch Environ Contam Toxicol. 2018 Sep 3;76(1):1–16. doi: 10.1007/s00244-018-0555-5 (PMC6326991; doi:10.1007/s00244-018-0555-5)
Supplement: Supplementary file 1 — Supplementary material 1 (DOCX 143 kb) [file 244_2018_555_MOESM1_ESM.docx]

**Funnel and Forest plots**


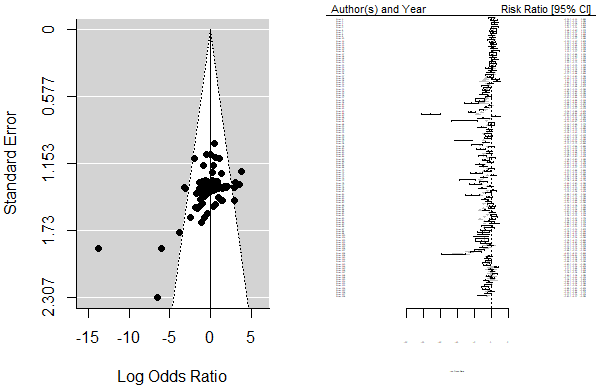


**Aqueous Cu/Reprouction**


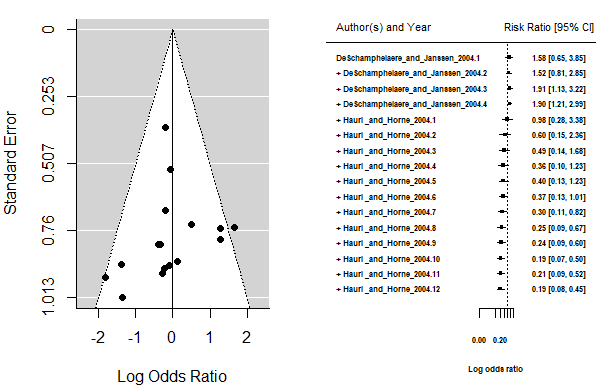


**Dietary Cu (< = 120 µg/L) /Reprouction**


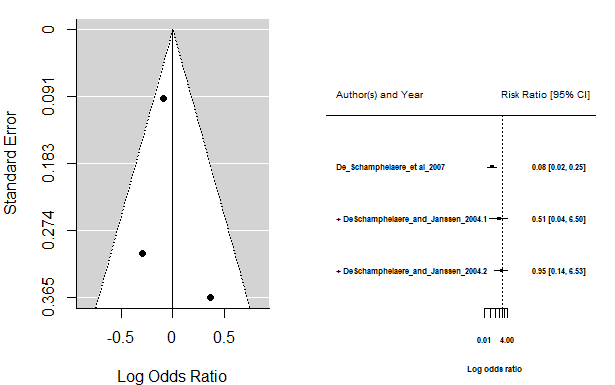


**Dietary Cu (>120 µg/L)/ Reprouction**


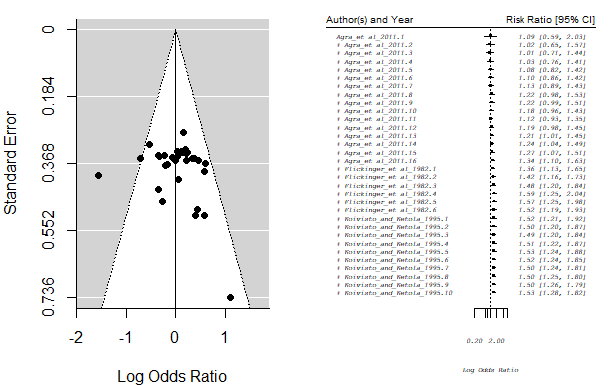


**Aqueous Cu/Age**


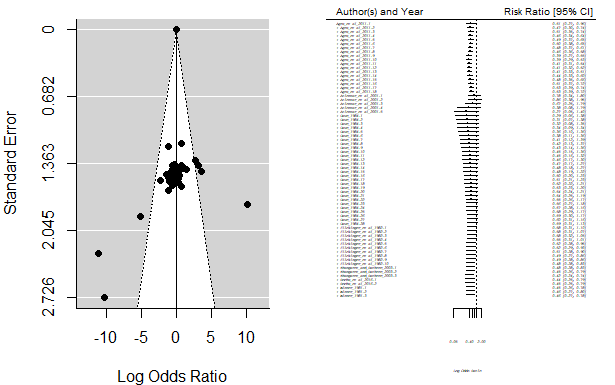


**Aqueous Cu/Growth**


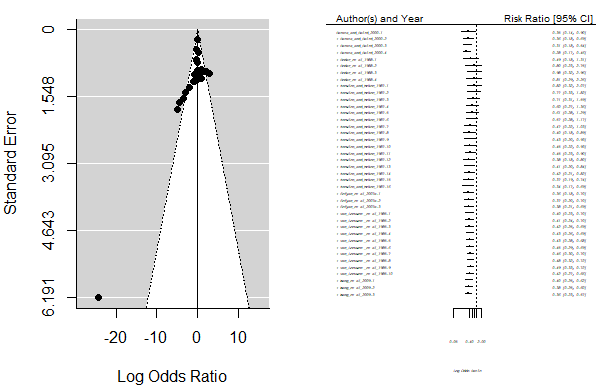


**Aqueous Cd (< = 40 µg/L) /Reprouction**


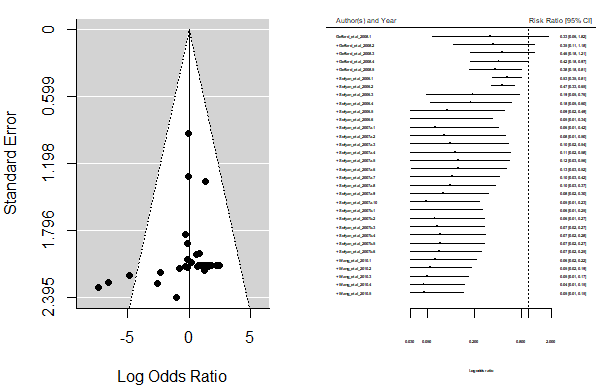


**Dietary Cd (> 40 µg/L) /Reprouction**


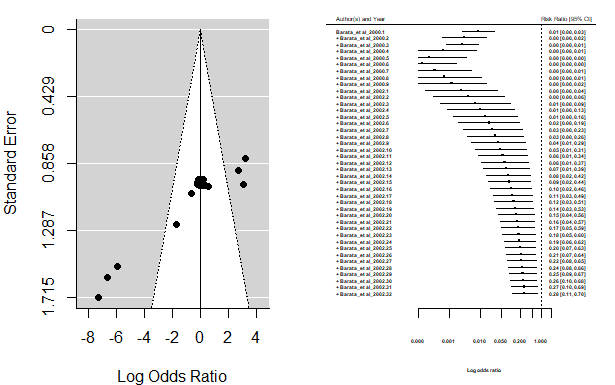


**Aqueous Cd /Age**


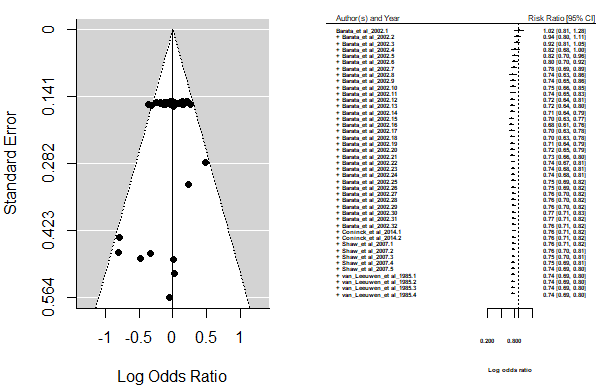


**Aqueous Cd /Growth**


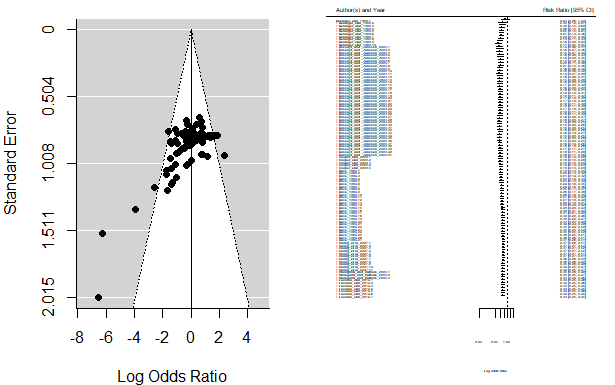


**Water hardness/ Cu**


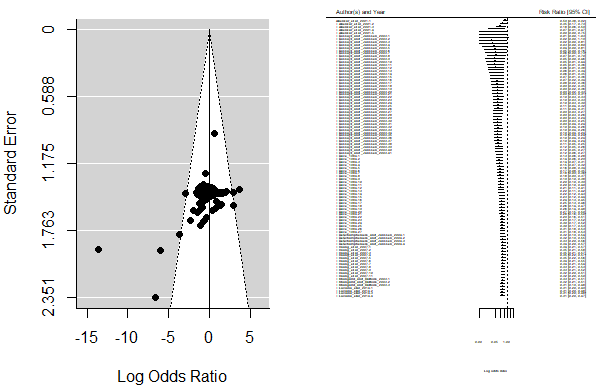


**Exposure Duration/Cu**


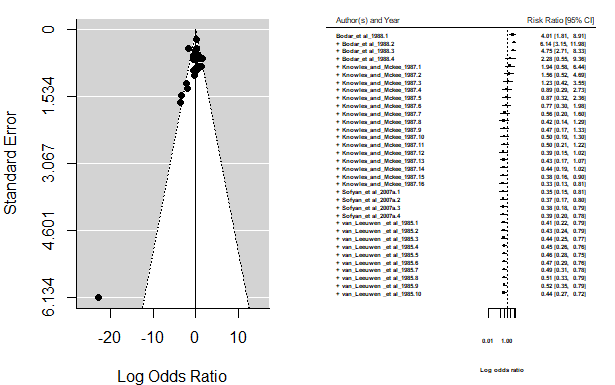


**Water hardness/ Cd**


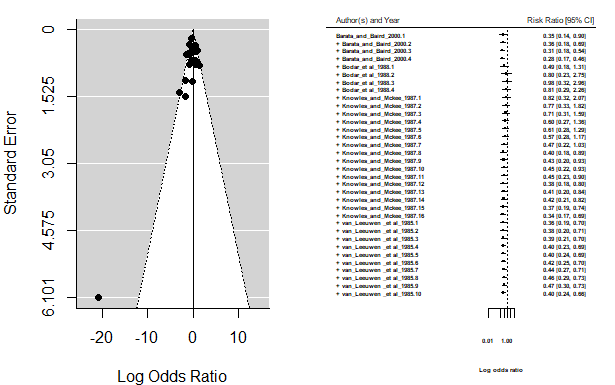


**Exposure Duration/ Cd**
